# Should I stay, or should I go: Modeling optimal flight initiation distance in nesting birds

**DOI:** 10.1371/journal.pone.0208210

**Published:** 2018-11-26

**Authors:** Liam Dowling, Frances Bonier

**Affiliations:** Department of Biology, Queen’s University, Kingston, ON, Canada; University of Tulsa, UNITED STATES

## Abstract

Flight initiation distance (FID)–the distance at which an individual leaves in response to the approach of a perceived threat–provides a measurement of risk-taking behavior. If individuals optimize their FID, this distance should reflect the point at which the fitness resulting from leaving exceeds the fitness resulting from all other possible decisions. Previous theory of FID has often been aimed at explaining this behavior in foraging individuals. Yet flight initiation in response to approaching threats occurs in a range of contexts that might influence optimal behavior. In breeding individuals, risk-taking decisions that are made at a location of offspring care (e.g., a nest or den) can have significant effects on fitness. Here, we develop a theoretical model of distances at which a parent bird flushes from a nest in response to an approaching threat. We estimate parent fitness with regards to characteristics of the parent (reproductive values, detection distance, and cost of lost parental care cost), the nest (concealment and accessibility), and the approaching predator (detection capability and predation success), developing a dichotomous scenario between staying at the nest or leaving at varying distances. Using a generalized comparison of the benefits of leaving versus staying, we find that increasing costs of lost parental care, probability of predation of the parent due to fleeing, or current reproductive value lead to more instances of staying at the nest. In a complementary approach with specified parameters based on biologically-informed factors that likely influence a predator-prey encounter, we find that increasing the current reproductive value, concealment of the nest, or costs of lost parental care decrease optimal FID and can lead to the parent staying at the nest. Other factors, such as increasing residual reproductive value, predation success, and predator capability of detecting the nest, increase optimal FID with some instances of costs of fleeing being so great that staying becomes an optimal strategy. Our theory provides a framework to explain variation in FID among nesting species and individuals and could provide a foundation for future empirical investigations of risk-taking behavior.

## Introduction

The way that prey respond to encounters with predators, like many other behaviors, is influenced by a balance of fitness benefits and costs [1]. Flight initiation distance (FID) is a common measurement that is thought to reflect the balance of this trade-off [2]. Simply defined, FID is the distance from an approaching predator at which the prey flees. FID is most often measured in foraging individuals, with lower values interpreted as reflecting increased risk-taking [2]. Yet flight initiation in response to approaching threats occurs in a range of contexts other than foraging, which might influence optimal behavior. For example, a meta-analysis of FID across taxa suggests that optimal decisions are made based upon multiple characteristics of the individual prey, the approaching predator, and the environment [3]. Risk-taking decisions that are made in parents attending offspring are complex, as these decisions involve fitness costs and benefits for the parent as well as their offspring. Evaluating how FID changes specifically in encounters at a nest may provide insight into the role that characteristics of the nest, parent, and approaching threat might have on this behavior.

An early theoretical model proposed by Ydenberg and Dill to explain FID examined the trade-off between costs and benefits associated with escape behavior [2]. This model proposed a simple break-even point between the cost of remaining and the cost of flight during a predator encounter. This economic model predicted that prey would flee from an approaching predator when the cost of remaining exceeded the cost of flight. This theory [2] was later expanded to include an optimization component as opposed to a simple break-even model [4]. This model generalized FID in terms of both benefits and costs gained during a foraging encounter as well as the probability of the prey surviving an attack (i.e., the perceived risk associated with the approaching threat). Another theoretical work to model FID examined how crypsis of prey influences the decisions of species during foraging [5].

Although these models provide a strong theoretical foundation to explain variation in FID, they cannot easily be applied to the context of parents attending offspring. Previous models focus on the direct cost to the individual, with no attention given to costs related to current reproductive efforts. This lack of acknowledgment of current reproduction is in contrast to life history theory, which predicts a trade-off between investment in oneself and one’s offspring, which could influence risk-taking decisions [6]. Notably, various empirical examples illustrate that an increased brood size relates to increased risk-taking behavior in parents when presented with a predator [1,7]. Specifically, longer FIDs (i.e., less risk-taking) were associated with smaller clutches among mallards (*Anas platyrhynchos*) and northern shovelers (*Spatula clypeata*) [8,9]. However, a comparative analysis of FID across 150 different avian species failed to find evidence of a general relationship between clutch size and FID [10]. This contrast likely reflects the fact that the comparative analysis assessed FID of birds that were not nesting (i.e., foraging or “relaxed behavior”) whereas the duck studies considered flushing distance of females from their nests where they were incubating clutches. As with current brood value, leaving the nest too readily may be associated with costs due to interruptions to incubation and other parental care. Lack of parental care from increased disturbance of the nest has been associated with increased nest failure rates [11,12]. In fact, throughout the incubation period, FID at nests has been found to decrease closer to the date of hatching of broods in mallards [9], greylag geese (*Anser anser*) [13], common goldeneyes (*Bucephala clangula*), and hooded mergansers (*Lophodytes cucullatus*) [14]. Thus, the consideration of current reproductive efforts in theoretical models may provide a more comprehensive understanding of FID in animals attending a nest.

A limitation of existing theories of FID is that they position the prey in a foraging or open-area context where the only option to ensure survival is fleeing. The haven of the nest may provide potential benefits to staying if it is well concealed or physically inaccessible to the predator. By leaving the nest, the parent may expose both themselves and the location of their brood to the predator. Previous theory and empirical evidences suggest that the cost of fleeing from a predator can increase as the FID decreases [5,15], and that optimal FID should decrease in cryptic species [3,16]. Although these examples consider the cost of self-exposure, they do not address the potential fitness costs of exposing a nest or brood. Two empirical studies that investigated nest coverage and concealment in five bird species reported that increased nest concealment was associated with decreased FID [8,17]. Along similar lines, three species of ground-nesting birds in Africa had decreased FIDs when their eggs and nests displayed high colour camouflage with the ground [18]. Thus, the physical environment of the encounter may play a pivotal role in the decisions that the parent makes when deciding to stay or flee.

Here, we draw from previous theoretical and empirical work to develop an original model that predicts optimal FID in parents attending a nest by examining various factors associated with the cost of exposure, cost of lost parental attendance at the nest, life history strategies, and the threat posed by the predator. This theory could provide a framework for future empirical research on risk-taking behavior in nesting species.

## Methods

### Establishing the model

To evaluate the optimal strategy of a parent, we develop a scenario that considers the potential outcomes and payoffs for a parent at a nest during an encounter with a predator. We use the specific scenario of a nesting bird confronted with an approaching predator to present our model, but the theory and its predictions could be generalized to apply to any animal that provides parental care in a localized area (e.g., a den or burrow). Within our model, we assume that the parent will only exhibit optimal behavior that maximizes their fitness. We make a novel assumption that not only does a parent have the choice of fleeing from the nest at a particular FID, but they may also decide to stay at the nest. We first compare the parent’s maximum expected fitness values between the two strategies (leave or stay) to determine an optimum behavioral decision.

We denote a potential payoff to the fitness of the parent to both their own survival and the survival of their offspring. A payoff of *f* is provided to the parent and represents their residual reproductive value. This value is proportional to the investment in self-maintenance and the expected number of offspring that parent will produce in the future. A payoff of *b* is given to the offspring and represents the current reproductive value. Tangibly, *b* can be estimated as the maximum number of offspring a parent has in their nest at the time of the encounter.

### Generalized Scenario

First, we examine a general scenario of a predator approaching a parent at a nest. The results from a generalized scenario provide insight into when leaving or staying at the nest is optimal and how variation in certain parameters, such as reproductive values, can lead to a shift between these alternate strategies. This method examines the transition between two strategies, but it does not estimate the distance of an optimal FID.

**Fig 1:**
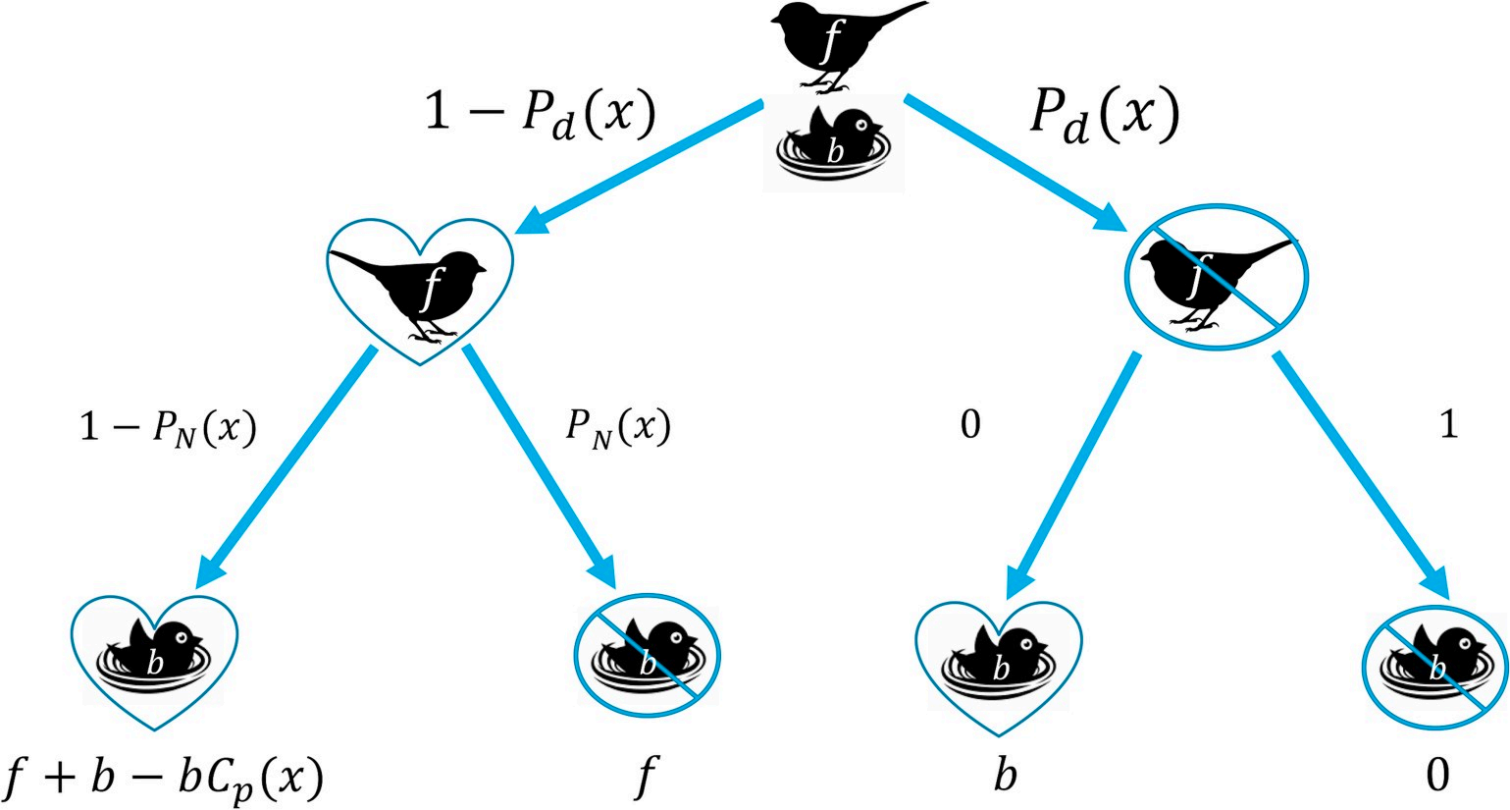
A decision tree of the relative fitness payoffs and probabilities of survival for the parent and nest based upon the decision to leave the nest as a function of flight initiation distance (FID). Hearts represent when an individual survives and circles with lines through them indicate when an individual does not survive. *f* represents the residual reproductive value of the parent and *b* represents the current reproductive value of the nest. The top branch point represents the probability of the parent dying from leaving (*P*_*d*_*(x)*) versus the probability of surviving (1- *P*_*d*_*(x)*). The left branch point represents the probability of the offspring dying (*P*_*N*_*(x))* versus surviving (1- *P*_*N*_*(x))*. The right branch point represents the probability of the offspring dying without any parental care, which we assume to be 1, or the probability of the offspring surviving without parental care, which we have set to 0. Values along the bottom of the tree represent potential payoffs from each scenario of *f* and *b* (as defined above), and *C*_*p*_*(x)* which reflects the cost of lost parental care for the current brood.

We begin by examining the potential payoff from leaving the nest at a particular FID, which we treat as the independent variable, *x*. The probability of the parent dying during the encounter is denoted by the function *P*_*d*_ (*x*) and consequently the survival of the parent would be 1 − *P*_*d*_ (*x*). In similar form, the probability of the eggs or young in the nest dying as a result of the encounter is represented as *P*_*N*_ (*x*) and the survival of the offspring is 1 − *P*_*N*_ (*x*). In combination, this provides four potential scenarios if the parent chooses to leave the nest: (1) both parent and offspring survive, (2) the parent survives and the offspring do not, (3) the parent dies and the offspring survive, and (4) both parent and offspring die (Fig 1).

For scenario 1 (the parent and offspring survive), the expected payoff will be the product of both survival probabilities multiplied by the payoff of *f* + *b*. For this decision, we have also included a fractional cost of *C*_*p*_*(x)* to the current reproductive value. This fraction must fall in the domain of 0 ≤ *C*_*p*_*(x)* ≤ 1 as the cost of lost parental care cannot exceed the current reproductive value. This value is a predicted cost associated with lack of parental care for the current offspring at the nest due to departure of the parent. For scenario 2 (the parent survives but the offspring do not), the fitness of the parent is only its residual reproductive value as it loses the fitness value from its current offspring. For scenario 3 (the offspring survive but the parents do not), we have denoted that there is a probability of 0 that the nest will survive if the parent dies. This assumption represents a natural biological scenario wherein offspring that do not have an attending parent are unlikely to survive throughout the rest of the nesting period. This assumption is reasonable for most species with altricial young but might not hold across all stages of nesting, or across species with a high degree of biparental care or those with more precocial young.

Taking all of these probabilities and payoffs into account, we arrive at an expected fitness payoff to the parent if they choose to leave the nest:

$$W_{L}(x)=[f+b-bP_{N}(x)-b(1-P_{N}(x))C_{p}(x)][1-P_{d}(x)],\;0\leq x\leq v$$ (1)

The fitness function exists on the domain between 0 and *v* where *v* is the distance at which the parent first detects the predator.

**Fig 2:**
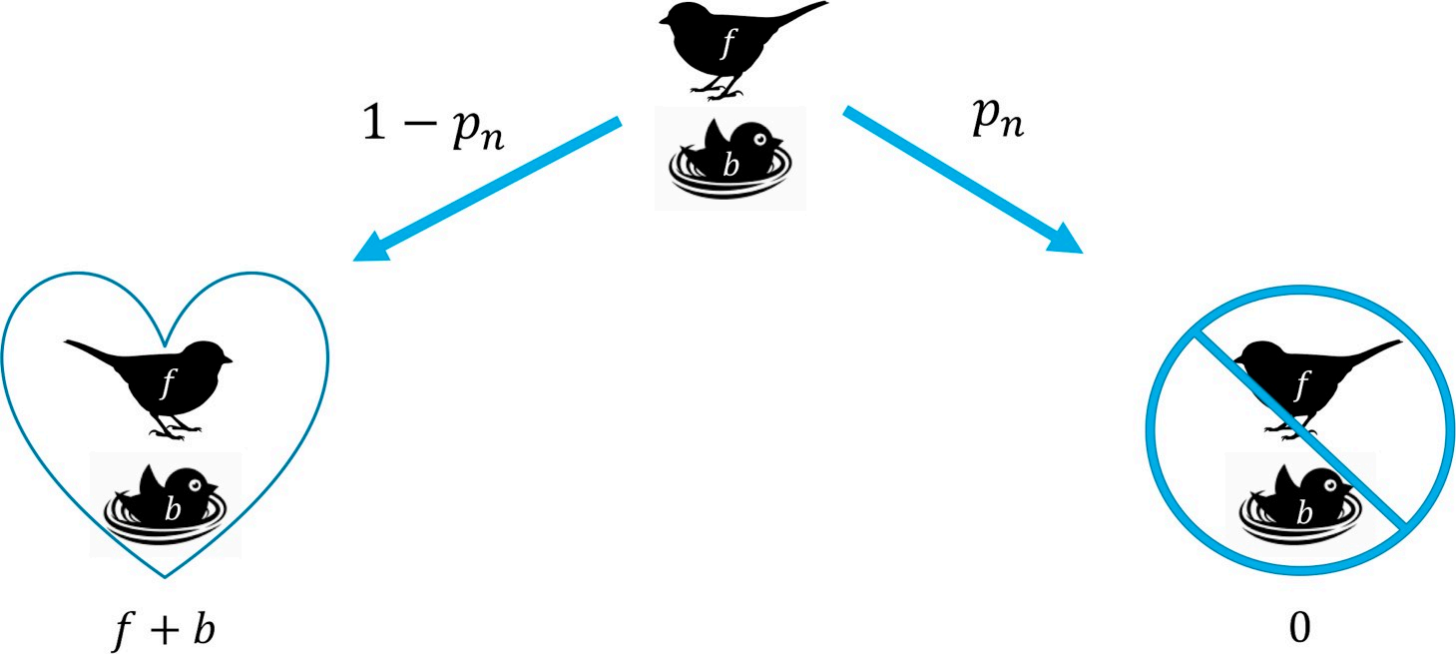
A decision tree of the relative fitness payoffs and probabilities of survival for the parent and offspring based upon the decision to stay at the nest. *f* represents the residual reproductive value of the parent and *b* represents the current reproductive value of the brood. Hearts represent when an individual survives and circles with lines through them indicate when an individual does not survive. The branch point compares the probability of the predator accessing the nest (*p*_*n*_) versus the predator not accessing the nest (1-*p*_*n*_). Payoffs are denoted along the bottom of the tree with the probability of a particular scenario occurring denoted along the decision arrow.

We next examine a general scenario of payoffs if the parent chooses to stay at the nest. Analyzing the decision to stay at the nest, we come to two potential options (Fig 2). We suppose that the probability that a predator is able to access the nest is *p*_*n*_ and conversely the probability of the nest being inaccessible is 1 − *p*_*n*_. The only potential payoff that a parent gains is in the situation where they survive the predator encounter (i.e., the predator does not access the nest).

From this situation, the expected payoff to the parent from choosing to stay at the nest can be summarized as:

$$W_{s}=[f+b][1-p_{n}]$$ (2)

where *W*_*S*_ does not represent a function of FID, but rather a specific constant value based on the ability of the predator to locate and access the nest.

For our analysis and results of this generalized scenario, we explore the conditions when the benefit from staying at the nest is greater than the benefit of leaving the nest (*W*_*S*_ *> W*_*L*_*(x)*). To provide a simple visualization of the outcome of this analysis we consider pairwise relationships between variables from the scenario. Because there are 6 variables of interest, there are a total of 15 possible pairwise comparisons, but for simplicity we only examine three of these combinations that share similar characteristics. The first pair is residual reproductive value (*f*) and current reproductive value (*b)* because these terms relate directly to a parent’s life history and are often functionally related to each other. The second pair is the probability of offspring death (*P*_*N*_*(x)*) and cost of lost parental care (*C*_*p*_*(x)*) as both of these terms relate to potential consequences to the offspring if the parent chooses to leave the nest. Finally, the last pair will be the probability of death to the parent from either leaving (*P*_*d*_*(x)*) or staying at the nest (*p*_*n*_) because both of these values directly relate to the survival of the parent. These two-dimensional planes allow us to visualize how the optimal strategy might shift with a change along any of these six axes, while all other variables are held constant. Extending further, we can manipulate the other four variables not displayed on the axes to examine how other parameters influence the optimality of one strategy, leaving or staying. We approach these results with the assumption that all factors can vary independently, although this may not be the case biologically.

### Specified Scenario

In addition to the generalized analysis described above, we also investigate the manner in which specific factors could potentially influence FID. Unlike the generalized scenario, this analysis provides insight into specific environmental factors that may influence FID. This analysis also provides insight into how the FID changes in length when leaving is an optimal strategy. To accomplish this specificity, we provide estimations for the generalized functions that reflect biologically plausible values. Our functions specify parameters for relevant environmental features that may play a role in influencing FID. The goal of this approach is to provide more refined predictions of risk-taking behavior of parents at nests based on factors such as nest structure and characteristics of a potential predator.

We define the probability of the parent dying after leaving the nest at a particular FID as:

$$P_{d}(x)=e^{\frac{-cx}{d}}\cdot e^{\frac{-x}{k}}$$ (3)

where *c* is the degree of concealment of the nest, *d* is the detection capability of the approaching predator, and *k* is the predation success rate of the predator. *c*, *d*, and *k* represent parameters within each individual function of *P*_*d*_*(x)* and demonstrate how changes in contextual factors can lead to changes in these individual probabilities. The first function represents the probability of the predator detecting the parent as it leaves the nest and can range from 0 and 1. A simple exponential probability was chosen as this represents an increasing likelihood of exposure as the distance between the parent and predator at the time of departure from the nest decreases. Notably, these parameters affect the probability of detection, where high levels of concealment (*c*) correlate with a faster rate of decay in detection probability at increased distances, while the inverse would be true for high levels of detection ability (*d*). We note the value of *d* cannot be 0 as there will always exist a non-zero probability that the predator will be able to detect the prey.

The second exponential function represents the probability that the predator successfully depredates the parent which also ranges from 0 to 1. We have similarly chosen an exponential function for probability of death as it represents a likely scenario of increased death as the predator comes closer to the nest. High values of predation success (*k*) will lead to an increase in the probability of death at a particular FID with the inverse also being true. We assume that *k* cannot be 0 as a predator will always have a non-zero probability of successfully killing a prey.

We have defined the probability of the offspring in the nest dying after the parent has left it unattended as:

$$P_{N}(x)=e^{\frac{-cx}{d}}\cdot p_{n}$$ (4)

where the first function is the probability of exposing the nest, as seen previously, and *p*_*n*_ is the probability of the predator physically accessing the nest. We assume a 100% predation rate if the predator is able to access the nest. This function represents a biologically realistic scenario of a predator having to both locate the nest and physically access it, so it would decrease in nests that are cryptic or well protected.

Finally, we have defined the cost of lost parental care from leaving the nest as a simple linear function:

$$C_{p}(x)=jx$$ (5)

where *j* is the fractional cost in terms of lost parental care due to leaving. We chose a linear function to represent the cost because individuals who leave at greater FID are assumed to spend longer periods off the nest and/or to react more frequently to perceived threats. We associate high values of *j* with broods that require a significant amount of parental care whereas low values would relate to more self-sufficient broods. For example, this value could decline as offspring develop thermoregulatory ability and are less reliant on parental brooding behavior.

We compare the maximum fitness value of leaving to the fitness value from staying to determine an optimal strategy. We further test how changes in particular parameters influenced the optimal FID and strategy, while holding all other variables constant. For our analysis, we use the following constant values, when looking at the influence of variation in other factors: *f* = 3, *b* = 3, *j* = 0.1, *c* = 5, *d* = 5, *k* = 5, *p*_*n*_ = 0.5 and *v* = 10. These values represent generic defaults and do not reflect a specific biological example, as our model provides a qualitative, as opposed to a specific quantitative assessment.

## Results

### Generalized Model

**Fig 3:**
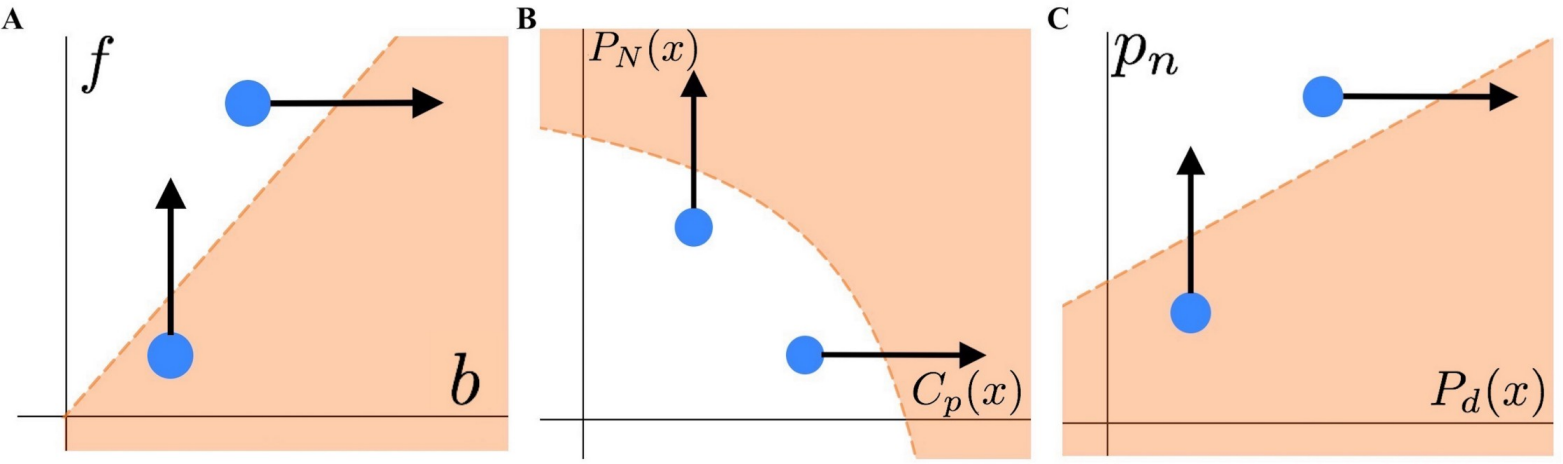
Shifting optimal strategies based on changing parameter and function values. For each panel, parameters are held constant except those represented on the two axes. The orange shaded regions illustrate scenarios where staying at the nest optimizes fitness (i.e., when *W*_*S*_ *> W*_*L*_*(x)*). The white regions illustrate scenarios where leaving the nest is the optimal strategy. The blue dot represents a random starting value with each arrow illustrating how optimal strategies change with increasing values along each axis. (A) The horizontal axis represents the current reproductive value (*b*) and the vertical represents the residual reproductive value (*f*). (B) The horizontal axis represents the cost of loss parental care (*C*_*p*_*(x)*) and the vertical axis represents the probability of offspring dying (*P*_*N*_ *(x)*). (C) The horizontal axis represents the probability of the parent dying from leaving (*P*_*d*_ *(x)*) and the vertical axis represents the probability of the parent dying from staying (*p*_*n*_).

By analyzing several pairwise relationships between the variables of interest, we find the range of optimal strategies that a parent can employ (Fig 3). We find that increasing values of residual reproductive value (*f*) lead to a transition from staying at the nest to leaving. Conversely, increasing values of current reproductive value (*b*) lead to a shift from leaving to staying at the nest (Fig 3A). When examining the effects of the other variables, if the probability of the parent dying from staying (*p*_*n*_) is less than the probability of the parent dying from leaving (*P*_*d*_*(x)*), the only optimal strategy will be to stay, regardless of the values of any other parameter. Leaving the nest can be the sole optimal strategy if the probability of death to the parent from leaving (*P*_*d*_*(x)*) is less than staying (*p*_*n*_) and there are relatively low consequences to the offspring from leaving (i.e. low values of *P*_*N*_*(x)* and *C*_*p*_*(x)*). A full range of changing optimal behavior can be explored in S1 Link.

We find that increasing the probability of the offspring dying (*P*_*N*_*(x)*) and the cost of lost parental care (*C*_*p*_*(x)*) leads to an optimal strategy of staying at the nest (Fig 3B). Examining the effect of the other parameters, again we find that if the probability of the parent dying from staying is less than leaving, the optimal strategy will be to stay regardless of any other parameter values. In order for leaving the nest to be the sole optimal strategy, the probability of death from leaving must be less than that for staying for the parent. If the probability of death from staying is greater than that for leaving, then reproductive values do not affect the optimal strategy. However, if the probability of death from staying is only marginally larger than leaving, a high ratio of residual to current reproductive value (i.e. *f* >> *b*) is needed for leaving to be the only optimal strategy. The specific effects that we describe can be examined in S2 Link.

Lastly, in response to an increased probability of death from fleeing (*P*_*d*_*(x)*), we find a transition from leaving to staying (Fig 3C). Conversely, we see that increasing the probability of death from staying (*p*_*n*_) leads to a transition from staying to leaving. We find that when there is no residual reproductive value (*f =* 0) and an extreme cost to the offspring (*P*_*N*_*(x)* or *C*_*p*_*(x)* = 1) the only optimal strategy will be to stay at the nest. All other possible combinations of the parameters will lead to both strategies being optimal depending on the ratio of the probability of death to the parent from leaving or staying. The effect of changing other parameters can be examined in S3 Link.

### Specified Model

**Fig 4:**
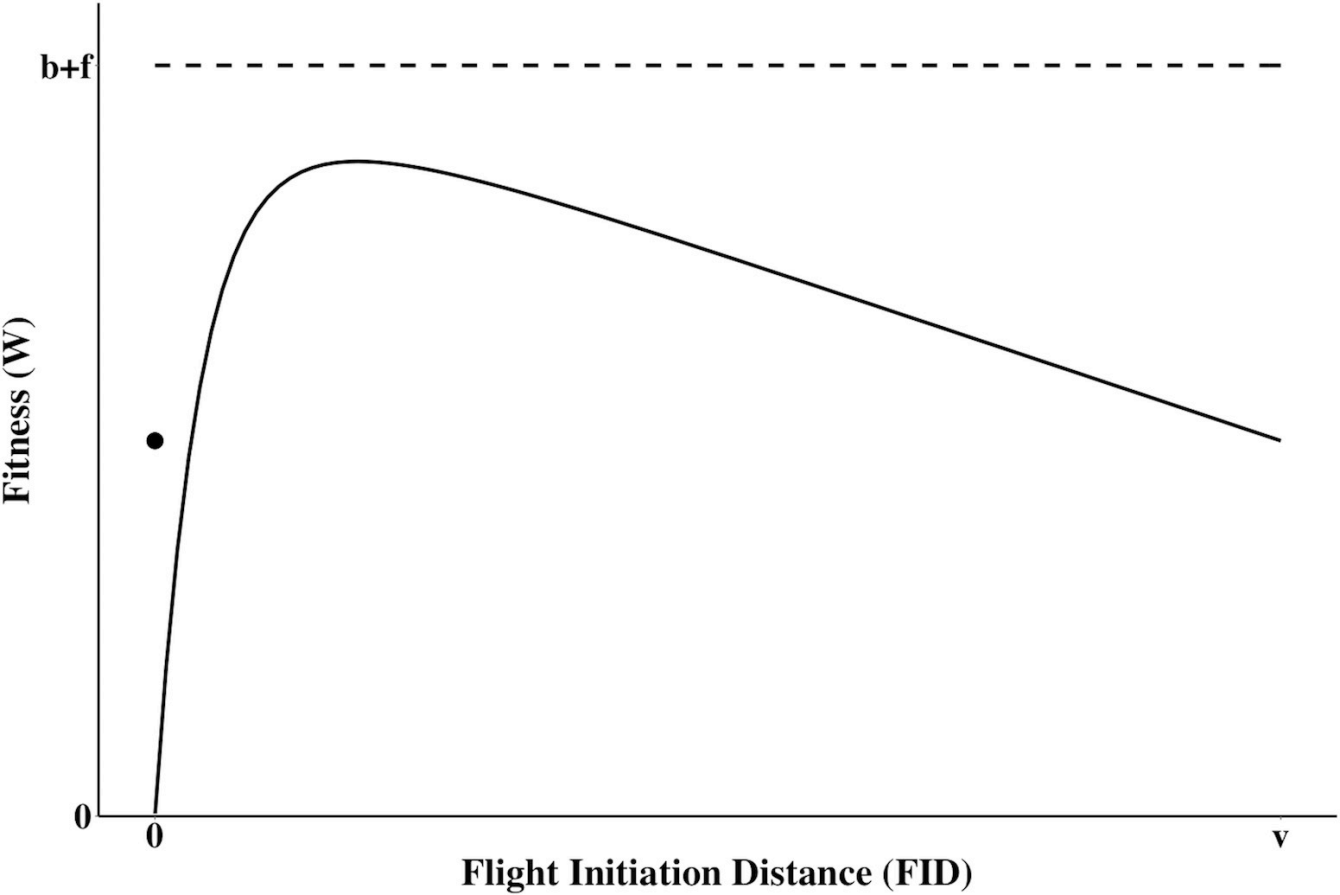
The expected fitness of the parent following the predator attack as a function of flight initiation distance (FID). *W*_*L*_(*x*) (Eq 1) defines the expected or average fitness after the attack if the parent chooses to leave (black line) relative to the fitness if a predator encounter does not occur (dashed line). The single point represents the expected fitness if the parent stays at the nest or *W*_*S*_ (Eq 2). *v* represents the distance at which the parent detects the oncoming predator. *b* + *f* represents the sum of current and residual reproductive value (i.e. parental fitness before the attack). The figure was derived using the default values of *f* = 3 (residual reproductive value), *b* = 3 (current reproductive value), *j* = 0.1 (cost of lost parental care), *c* = 5 (nest concealment), *d* = 5 (predator’s detection capability), *k* = 5 (predation success), *p*_*n*_ = 0.5 (nest accessibility), and *v* = 10 (detection distance).

By assigning generic default values for each of our parameters, we see the relative fitness of a parent from leaving the nest at a certain FID or choosing to stay at the nest throughout the encounter (Fig 4). The specified model predicts a situation in which individuals who experience an attack will have, on average, a reduction in fitness after the event regardless if they choose to leave or stay. Given this scenario, with constant parameter values, there exists some optimal FID located on the domain 0 ≤ *x* ≤ *v*.

**Fig 5:**
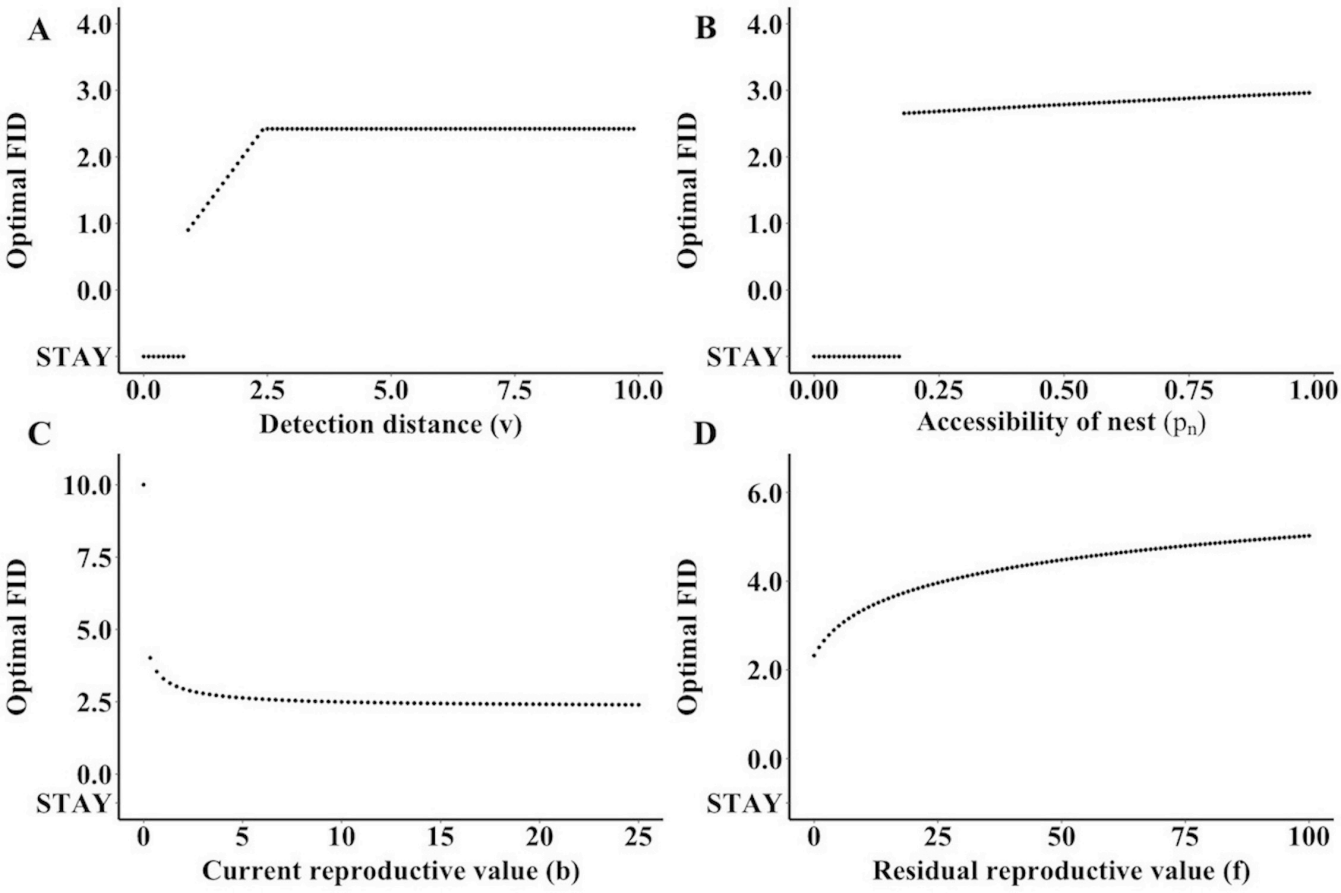
**Changes in optimal FID as a result of variation in ecological parameters: (A) detection distance (*v*), (B) accessibility of the nest (*p***_***n***_**), (C) current reproductive value (*b*), and (D) residual reproductive value (*f*).** Each point represents the optimal strategy of FID for the parent as a predator approaches for a discrete parameter value. The figure was derived using the default values of *f* = 3 (residual reproductive value), *b* = 3 (current reproductive value), *j* = 0.1 (cost of lost parental care), *c* = 5 (nest concealment), *d* = 5 (predator’s detection capability), *k* = 5 (predation success), *p*_*n*_ = 0.5 (nest accessibility), and *v* = 10 (detection distance).

The optimal FID for a parent varies with the distance at which it detects the oncoming predator, *v* (Fig 5A). The predicted optimum shifted down along with the detection distance until it reached a threshold where the fitness from staying exceeded all other fitness values.

Changes in nest accessibility, *p*_*n*_, lead to a piecewise evaluation of optimal FID (Fig 5B). The specific value of the threshold between the optimal strategy shifting from leaving to staying at the nest depends on the value of other parameters, but regardless it forms a strict dichotomy between types of nesting protection. At low values of nest accessibility, the optimal behavior is to stay in the nest. However, above a threshold, the optimum shifts to a non-zero value and from there continually increases until the values stop at the upper limit of *p*_*n*_ = 1, when the nest is completely accessible to the predator.

The current reproductive value, *b*, significantly alters the optimal FID (Fig 5C). When current reproductive value is 0, the optimal FID is at the detection distance. As *b*, increases, the optimum FID shifts closer to the nest, eventually plateauing at high values of *b*. In contrast, increasing residual reproductive value leads to longer FIDs and the values do not appear to have an upper limit (Fig 5D). Even when the parent has a residual reproductive value of 0, the optimum is at a non-zero FID.

**Fig 6:**
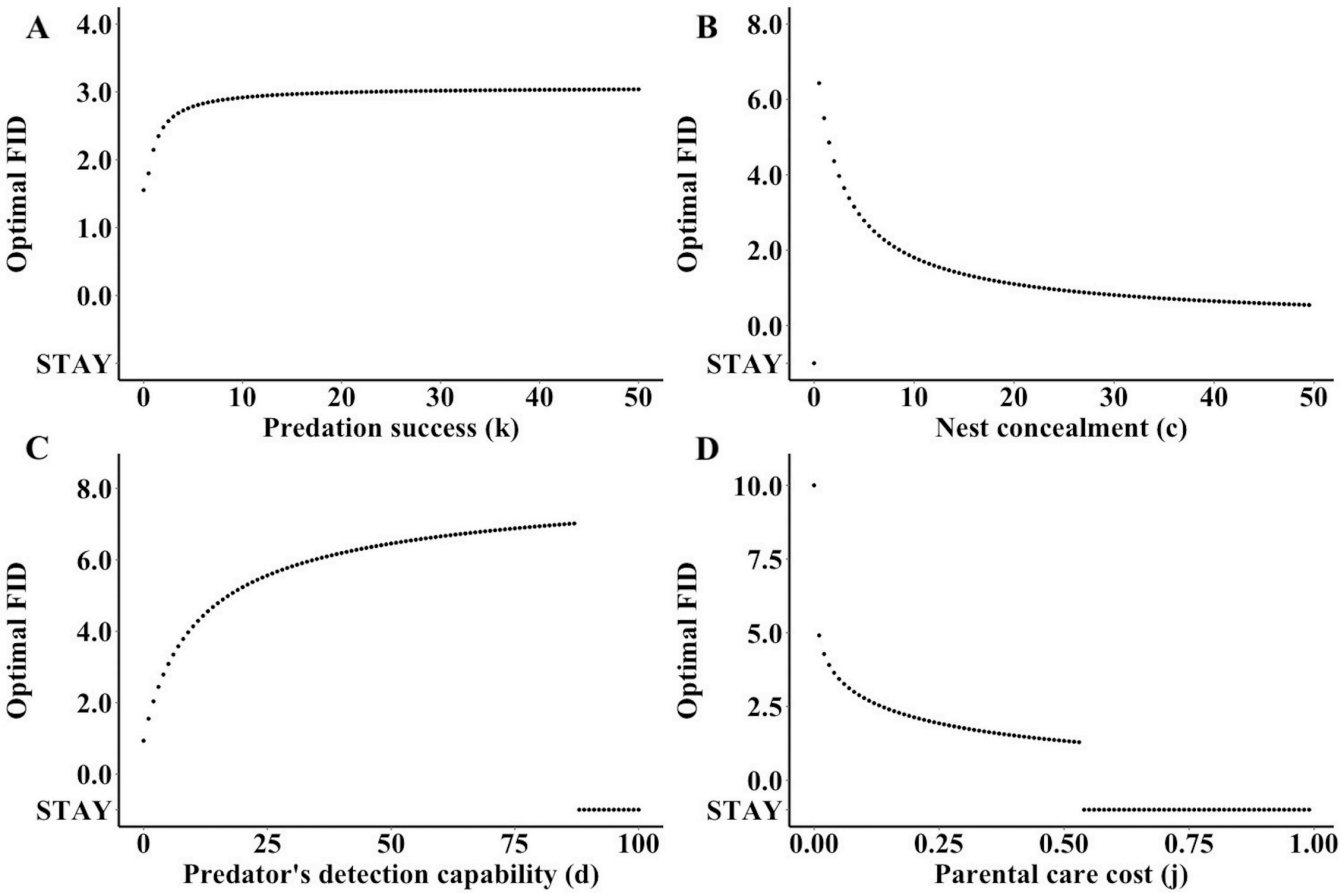
**Changes in optimal FID as a result of variation in ecological parameters: (A) predation success (*k***)**, (B) nest concealment (*c*), (C) predator detection capability (ability to detect the parent as it leaves the nest) (*d*), and (D) cost of lost parental care *(j*).** Each point represents the optimal strategy of FID for the parent as a predator approaches for a discrete parameter value. The figure was derived using the default values of *f* = 3 (residual reproductive value), *b* = 3 (current reproductive value), *j* = 0.1 (cost of lost parental care), *c* = 5 (nest concealment), *d* = 5 (predator’s detection capability), *k* = 5 (predation success), *p*_*n*_ = 0.5 (nest accessibility), and *v* = 10 (detection distance).

An increase in the probability that the predator successfully depredates the parent (*k*) leads to a longer optimal FID (Fig 6A). Eventually, the optimum decelerates, appearing to plateau at large values of *k*.

The effect of nest concealment on the choice of the parent is bifunctional (Fig 6B). For nests with a low level of concealment, *c*, the optimal strategy FID is staying at the nest approaches the nest. A slight increase in concealment shifts the optimum strategy to a long FID. Finally, as concealment continues to increase, the optimal FID eventually decreases to a smaller, yet still non-zero optimum.

The detection capability of the predator displays a similar split functional nature, but with the opposite trend similar trend to nest concealment (Fig 6C). Low levels of detection capability result in a shorter optimal FID, but as the detection skill increases, so does the optimal FID to a maximum. After this point, optimal FID begins to return back to the nest. This trend continues until a threshold is reached when the optimum shifts back to an optimal strategy of staying at the nest.

When there is no cost of lost parental care (i.e., *j* = 0), optimal FID is at the detection distance and, as the cost increases, the optimal FID continually decreases (Fig 6D). There exists a point when the gradual decrease of optimal FID abruptly shifts to a stable optimal strategy of staying at the nest.

## Discussion

We developed a theoretical framework to examine how the optimal behavior of individuals attending a nest changes with varying characteristics of the individual, the nest, and the predator. Our two approaches produced complementary results yet provide different insights into this behavior. The generalized model provides a framework to understand factors that might cause an individual to switch between the strategy of staying at or leaving the nest during a predator encounter. Although this model does not provide specific insight into how optimal FID changes, these results suggest how an individual’s general strategy may change in response to a change in their environment. Our specified model relied on assumptions of how a predator may detect and depredate an individual, and it provides insights into how variation in environmental factors may influence optimal FID. This model also allows consideration of how optimal FID might vary with changing parameters, as it was not constrained to a dichotomous choice.

Our generalized results suggest that increasing probability of offspring death, current reproductive value, probability of parent death from leaving, and cost of lost parental care, while holding all other parameters constant, lead to more instances of staying at the nest (Fig 3). However, increases in residual reproductive value and probability of parent death from staying lead to the parent leaving the nest. We found that switching to a different strategy is highly dependent on the parent’s probability of death. Changes in these environmental and contextual factors theoretically lead a parent to change their behavior in response to predators.

Our specified results predict that parents that are unable to detect approaching predators at far distances may have sub-optimal FIDs compared to individuals with a greater detection range. Accessibility of the nest caused a piecewise split between staying and non-zero optimal FID at a particular threshold (Fig 5B). We also estimated piecewise splits for nest concealment, predator detection capability, and cost of lost parental care (Figs 5 and 6). Optimal FID increased continuously with predation success, until staying became an optimal strategy (Fig 6A). Related to life history strategies, increases in current reproductive value lead to shorter FIDs (Fig 5C), whereas larger residual reproductive values lead to longer FIDs (Fig 5D). Although we evaluated these variables shifting independently, some variables such as current and residual reproductive value, and nest accessibility, nest concealment, and detection distance may be functionally linked with each other in nature. Additionally, the model assumes that there is no effect of previous exposure to predators on optimal FID. It has been demonstrated both empirically and theoretically that prior knowledge of predators can influence the risk-taking behaviour of an individual [3,19]. Previous predator encounters could increase the cost to parental care (*j*) due to repeated departures, leading to an optimal strategy of staying at the nest more frequently because of accumulated costs. Past experiences could also increase the detection distance (*v*) and allow parents to more accurately estimate the threat posed by the approaching predator (*k*), which could lead to habituation and fewer instances of departure from the nest or shorter FID.

A novel aspect of our theory was the inclusion of the effect of current reproductive value of the brood on optimal FID. Life history theory suggests that a higher investment in current reproduction should lead to increased risk-taking behavior, when it might benefit offspring survival [1,6,7], which is consistent with both our generalized and specific scenario results (Fig 5C). The introduction of a non-zero current reproductive value led to a dramatic shift of more incidents of staying or optimal FID closer to the nest. Ydenberg and Dill’s break-even model suggests that increased costs of leaving should lead to shorter FIDs [2], but our model specifically incorporates costs based on current reproductive value. This result is consistent with nest defence theory [1,20] as well as some empirical evidence that large clutch sizes correlate with shorter FIDs from the nest [8,9].

As predicted by life history theory, we see that an increased residual reproductive value leads to more instances of leaving the nest and greater FIDs, i.e., less risk-taking behavior (Fig 5D). In their theory of optimal FID during foraging, Cooper and Frederick predicted that higher residual reproductive value would also lead to longer FIDs [4]. This relationship suggests that residual reproductive value may act as a strong driver of risk-taking behavior across many environmental contexts. Empirical evidence to support this theory comes from duck species, where a lower annual mortality is associated with longer FIDs and generally less risk-taking behavior [9]. Similarly, in a comparative study examining FID across over 150 species of birds, species with a greater body mass exhibited longer FIDs [10]. Given the general positive association between body size and annual survival [21], this finding also agrees with the predictions of our model.

The trade-off between current and residual reproductive value in relation to risk taking proposes an evolutionary explanation for differing behavior in nesting species. Our results suggest that individuals with slow life history strategies will optimally display less risky behavior, because higher residual reproductive values were associated with increased FID. However, those individuals with fast life histories will be more likely to display risky behavior as we showed increasing the current reproductive value was associated with reduced FID. Ample empirical evidence supports this theory [1,7,10,22–24].

The ability of a parent to detect an approaching predator can provide potential fitness benefits to the individual. From our model, we see that limited sightlines or otherwise constrained detection abilities could lead to a sub-optimal strategy (Fig 5A). This sub-optimal strategy is consistent with previous work on nest concealment trade-offs, which find that nesting birds might not maximize nest protection in order to maintain environmental sensory information [25]. In fact, high levels of protection of the nest are directly correlated with visual limitations with only one to four percent of visible light entering some nest cavities [26,27]. A study of rabbits hiding in vegetation directly examined how percent visibility from the prey’s perspective related to FID [28]. These results describe a similar pattern to that predicted by our theory, with individuals exhibiting longer FIDs as visibility increases until values reach a plateau. Although low visibility can sometimes lead to sub-optimal strategies, it can also potentially lead to optimal strategies if nest accessibility is also low. This expectation occurs because low levels of nest accessibility naturally shift the optimal strategy to staying at the nest. We would therefore expect that individuals would attempt to maximize their ability to detect approaching predators, except in cases where high levels of physical protection from predation beneficially trade off with losses in detection ability.

The probability of a predator physically accessing a nest (*p*_*n*_) plays a significant role in determining the optimal strategy for the parent. In our generalized model, high values of *p*_*n*_ were associated with a stable optimal strategy of leaving the nest. With the specified model, low values of *p*_*n*_ shift the optimal strategy to staying at the nest (Fig 5B), suggesting that many cavity-nesting species would be unlikely to leave the nest during an encounter, assuming they can assess the predator’s ability to access the nest. A meta-analysis of fear in animals demonstrated that FID increased with increasing distance to a refuge [3]. Interestingly, the piecewise shape of the optimal FID predicted by our model suggests that a species’ nesting ecology can influence selection on flushing behavior, leading to drastically different strategies among species.

The last variable directly related to the probability of survival of the parent was relative predation success, *k*. Increased probability of death pushes the optimum FID further away from the nest (Fig 6A). This theory directly supports the previous theoretical work on FID during foraging encounters, which predicted that greater costs associated with staying should lead to increased FIDs [2,4]. Different predators should therefore lead to varying optimal strategies. Notably, a meta-analysis showed that the size of predators significantly predicted FID, with longer FIDs associated with larger predators [3]. We can conclude that individuals that are subject to encounters with a variety of different predators that are able to adjust their escape strategy accordingly would be at a fitness advantage compared to individuals with a static strategy.

We found that high levels of concealment or crypsis were associated with decreased FID, which is consistent with empirical examples [3,8,17,18]. However, we found that the optimum strategy shifts to staying at the nest with low values of concealment or high values of predator detection capability (Fig 6B and Fig 6C). Examining the shape of the probability of detection function can explain this strategy shift. When the ratio between concealment (*c*) and detection capabilities (*d*) becomes relatively low, the probability of detection is approximately the same across all FIDs. Therefore, the parent will have an optimal strategy of staying at the nest as this choice minimizes both the exposure cost and the cost from lost parental care. Although some studies have investigated the effect of different types of predators approaching a nest on risk-taking behavior [29], additional empirical work directly related to how the detection capability of an approaching predator influences FID is needed.

Lastly, the cost of lost parental care shifted optimal FIDs closer to the nest until reaching a threshold beyond which staying at the nest was the optimal strategy (Fig 6D). Our results are consistent with other studies that have suggested that increased disturbance leads to increased nest failure and consequently an increased fitness cost [11,12]. During critical periods of parental care, the value of lost care (*C*_*p*_*(x)* or *j*) will be high due to flushing from the nest. We might expect these costs to be high during incubation and immediately following hatching, when offspring are highly dependent on parental care. Conversely, there may be lower parental care costs during the late stages of breeding when offspring are more self-sufficient. For example, in greylag geese, FIDs decreased closer to hatching dates [13]. Along similar lines, across-species analyses in bird species report lower FIDs from the nest associated with later stages of incubation [9,14]. However, there is a need for research investigating how FID changes throughout a breeding period, and across species with varying degrees of offspring dependence on parental care.

Our findings suggest that even small changes in breeding ecology may sometimes have significant consequences in terms of risk-taking behavior both among species, populations, and individuals. Our model presents a novel incorporation of current reproductive value and a choice between leaving or staying and could ultimately provide testable hypotheses for further investigations into FID behavior in nesting birds and breeding animals more generally.

## Supporting information

S1 Link Graphical software demonstrating the optimal strategy of a parent based on changing residual (*f*) and current (*b*) reproductive values. Here we provide a link to a *Desmos* plane that shows all possible combinations of reproductive values. Orange space represents when the optimal strategy is to stay at the nest and white space represents when the optimal strategy is to leave the nest.
(DOCX)

S2 Link Graphical software demonstrating the optimal strategy of a parent based on changing probability of offspring death (*P*_*N*_*(x)*) and cost from lost parental care (*C*_*p*_*(x)*). Here we provide a link to a *Desmos* plane that shows all possible combinations of the two functions associated with sequences to the nest from a parent leaving. Orange space represents when the optimal strategy is to stay at the nest and white space represents when the optimal strategy is to leave the nest.
(DOCX)

S3 Link Graphical software demonstrating the optimal strategy of a parent based on changing probabilities of death to the parent from staying (*p*_*n*_) and leaving (*P*_*d*_*(x)*) the nest. Here we provide a link to a *Desmos* plane that shows all possible combinations of probability of death to the parent. Orange space represents when the optimal strategy is to stay at the nest and white space represents when the optimal strategy is to leave the nest.
(DOCX)

S4 Link Graphical software to evaluate changes in the fitness functions resulting from a change in the various parameters. Here we provide a link to a *Desmos* graph where all of the functions and parameters are listed. Individual sliders can be adjusted to visualize the change in optimum flight initiation distance strategy.
(DOCX)
