# Supplementary material for: Should I stay, or should I go: Modeling optimal flight initiation distance in nesting birds
Source: PLoS One. 2018 Nov 26;13(11):e0208210. doi: 10.1371/journal.pone.0208210 (PMC6258376; doi:10.1371/journal.pone.0208210)
Supplement: S2 Link — Here we provide a link to a Desmos plane that shows all possible combinations of the two functions associated with sequences to the nest from a parent leaving. Orange space represents when the optimal strategy is to stay at the nest and white space represents when the optimal strategy is to leave the nest. (DOCX) [file pone.0208210.s002.docx]

S2 Link

<https://www.desmos.com/calculator/6uv2ct55lw>
