# Supplementary material for: Should I stay, or should I go: Modeling optimal flight initiation distance in nesting birds
Source: PLoS One. 2018 Nov 26;13(11):e0208210. doi: 10.1371/journal.pone.0208210 (PMC6258376; doi:10.1371/journal.pone.0208210)
Supplement: S3 Link — Here we provide a link to a Desmos plane that shows all possible combinations of probability of death to the parent. Orange space represents when the optimal strategy is to stay at the nest and white space represents when the optimal strategy is to leave the nest. (DOCX) [file pone.0208210.s003.docx]

S3 Link

<https://www.desmos.com/calculator/g21umyaz7u>
