# Supplementary material for: Should I stay, or should I go: Modeling optimal flight initiation distance in nesting birds
Source: PLoS One. 2018 Nov 26;13(11):e0208210. doi: 10.1371/journal.pone.0208210 (PMC6258376; doi:10.1371/journal.pone.0208210)
Supplement: S4 Link — Here we provide a link to a Desmos graph where all of the functions and parameters are listed. Individual sliders can be adjusted to visualize the change in optimum flight initiation distance strategy. (DOCX) [file pone.0208210.s004.docx]

S4 Link

<https://www.desmos.com/calculator/i6b9xplolq>
